# Treatment of White Swelling

**Published:** 1847-02

**Authors:** 


*Treatment of White Swelling.* Dr. Blakely details two cases of the successful employment of the *Chimaphila Umbellata* (pipsissewa, wintergreen) in the treatment of white swelling. The first case, a boy of a scrofulous famly, is thus described:

When I examined my patient, I found one of his knees three times its natural size, the skin of the leg of an unnatural ashy color, the boy being tolerably black for one of his race; considerable wasting of the limb, pulse 96, and some white fur upon his tongue. I looked upon the case as scrofulous white swelling, and concluded in my own mind there could be little done towards effecting a radical cure, as I had often treated and seen such cases treated, but had never known a cure to follow, but more or less lameness to inevitably succeed all our efforts, if we did not ultimately have to resort to the knife to rescue the sufferer from the grave.

I commenced giving my patient the infusion of pipsissewa, a pint to be drank each day. The formula for making it I took from Wood and Bache's Dispensatory, and twice a day, morning and night, I had a fresh poultice made out of oat-meal and the infusion, and applied to the whole knee; diet light, and to keep the recumbent position.

The treatment was continued from February 5th to April 15th, when the patient was discharged cured.—*Southern Medical and Surgical Journal.*

